# High-Throughput Study of the Effects of Celastrol on Activated Fibroblast-Like Synoviocytes from Patients with Rheumatoid Arthritis

**DOI:** 10.3390/genes8090221

**Published:** 2017-09-06

**Authors:** Zhengyu Fang, Dongyi He, Bo Yu, Feng Liu, Jianping Zuo, Yuxia Li, Qi Lin, Xiaodong Zhou, Qingwen Wang

**Affiliations:** 1 Biomedical Research Institute, Shenzhen Peking University-The Hong Kong University of Science and Technology Medical Center, Shenzhen 518036, China; fangzy796@163.com (Z.F.); yuxiali2014pku@163.com (Y.L.); 2 Department of Rheumatism and Immunology, Peking University Shenzhen Hospital, Shenzhen 518036, China; liufeng2127@163.com (F.L.); linqi0121@126.com (Q.L.); 3 Guanghua Integrative Medicine Hospital, Shanghai 200052, China; hedongyish@163.com; 4 Department of Dermatology, Peking University Shenzhen Hospital, Shenzhen 518036, China; yubomd@163.com; 5 Shanghai Institute of Materia Medica, Chinese Academy of Sciences, Shanghai 201203, China; jpzuo@mail.shcnc.ac.cn; 6 Department of Internal Medicine, The University of Texas Health Science Center at Houston, Houston, TX 77030, USA

**Keywords:** celastrol, rheumatoid arthritis, fibroblast-like synoviocyte, chemokine, NF-κB signaling pathway

## Abstract

Celastrol, a natural triterpene, exhibits potential anti-inflammatory activity in a variety of inflammatory diseases. The present study aimed to investigate its biological effect on activated fibroblast-like synoviocytes (FLSs) from patients with rheumatoid arthritis (RA). The primary FLSs of the synovial tissues were obtained from synovial biopsies of patients with RA. The normal human FLS line (HFLS) was used as a control. After the RA–FLSs and HFLSs were treated with or without celastrol, various approaches, including the WST-1 assay, transwell assay, real-time PCR and ELISA analysis, were performed to estimate proliferation, invasion and expression of pro-inflammatory cytokines of the RA–FLSs. Microarray analysis was performed to screen for differentially expressed genes in RA–FLSs before and after celastrol treatment. The results showed that treatment of celastrol attenuated both the proliferation and invasion of the RA–FLSs. The expression of several chemokine genes, including *CCL2*, *CXCL10*, *CXCL12*, *CCR2* and *CXCR4*, was significantly changed after celastrol treatment. The genes involved in the NF-κB signaling pathway appeared to be regulated by celastrol.

## 1. Introduction

Rheumatoid arthritis (RA) is a chronic inflammatory disorder that affects multiple peripheral joints [1,2]. It is characterized by synovial hyperplasia, which results in joint destruction [3,4]. One of the main cell types involved in the pathological process of RA joint tissue are the fibroblast-like synoviocytes (FLSs). In the progression of RA, FLSs grow in an anchorage-independent fashion with changes in morphology, and they are considered to be in an activated status as they strongly express some biomarkers such as c-fos, Jun-B, egr-1 and MMP3 [5,6,7]. The mechanisms involved in the development of this phenotype are unclear, although some reports implicate an association with epigenetics [8,9]. In addition, FLSs can promote various processes in RA by secreting different types of inflammatory cytokines, such as IL-6, IL-8, IL-1β, TNF-α and MCP-1, as well as matrix metalloproteinases (MMPs), such as MMP-1 and MMP-13 [10,11,12,13].

Celastrol, a natural triterpene, is an active ingredient isolated from *Tripterygium wilfordii Hook f* (TWHF) [14,15]. The latter has been widely used in the treatment of RA, autoimmune disease and inflammatory disease in China [16]. It can evidently improve the symptoms and laboratory indicators of RA. Although the clinical efficacy of celastrol has been well-documented, its basic mechanism of action remains unclear. Previous in vitro studies have shown that high doses of celastrol could induce apoptosis and inhibit growth and invasion of RA [17,18]. However, there is no high-throughput study on the influence of celastrol treatment on activated FLSs from RA patients.

In the present study, we aimed to investigate the gene expression profiles regulated by celastrol in the activated FLSs from RA patients. The results indicated that celastrol could influence the expression levels of several chemokines and chemokine receptors in RA–FLSs. The NF-κB signaling pathway was partially involved in this progress.

## 2. Materials and Methods

### 2.1. Isolation and Primary Culture of RA–FLSs

This study was approved by the Ethics Committee of Peking University Shenzhen Hospital (IRB00001026-07632). Synovial tissues were obtained from biopsies of two patients fulfilling the American Rheumatism Association criteria for RA [19]. All patients were informed about the aims of specimen collection and gave signed written consent in accordance with the ethical guidelines of Peking University. The tissue was placed in cell culture medium at ambient temperature and subjected to tissue digestion within 2 h. Synovectomy samples of RA were finely minced, digested for 30 min at 37 °C in phosphate-buffered saline (PBS) containing 0.1% trypsin (Sigma, Deisenhofen, Germany), and thereafter digested in 0.1% collagenase P (Boehringer Mannheim, Mannheim, Germany) in Dulbecco’s modified Eagle medium (DMEM)/10% fetal calf serum (FCS) for 2 h at 37 °C, 5% CO_2_. The cell suspension was then filtered, and the cells were collected by centrifugation. Cells were kept in primary culture for seven days (DMEM/10% FCS, 25 mM HEPES, 100 U/mL penicillin, 100 µg/mL streptomycin and 2.5 µg/mL amphotericin B (Sigma, St. Louis, MO, USA), including removal of non-adherent cells on days 1, 3, 5 and 7) and subsequently used for FLS isolation. The samples were randomly tested to exclude mycoplasma contamination. For negative isolation of SFB from primary culture, adherent synovial cells were detached by short-term trypsinization for 2 min (0.25% trypsin/0.2% EDTA; Gibco, Gaithersburg, MD, USA) and 10^7^/mL synovial cells were incubated with 4 × 10^7^/mL Dynabeads^®^ M-450 CD14 (clone RMO52; Dynal, Hamburg, Germany) in PBS/2% FCS for 1 h at 4 °C. Nine milliliters of PBS/2% FCS were then added, and the conjugated cells were collected using the Dynal magnetic particle concentrator. These cells as well as the normal human fibroblast-like synoviocyte (HFLS, Cell Application, Catalog #408-05a) line were all cultured using Synoviocyte Medium (SM, Sciencell, Carlsbad, CA, USA, Catalog #4701), which contained fetal bovine serum and synoviocyte growth supplement.

### 2.2. Immunofluorescence Staining

After the isolated fibroblast-like cells climbed and spread to the carry sheet glass, they were fixed by 2% paraformaldehyde for 15 min, and the coverslips were blocked by rabbit serum (Sigma) for 1 h, followed by incubation with anti-Vimentin (1:50, Abcam, Cambridge, MA, USA) antibody for 1 h. After washing with 0.01% saponin in PBS 3 times for 15 min each, the coverslips were incubated with secondary antibody conjugated with fluorescein (FITC, Jackson Immuno Research, West Grove, PA, USA) for another hour. The slips were then washed and DAPI solution was used for nuclear stain. The coverslips were further washed with 0.01% saponin in PBS, three times, 15 min each, and PBS twice, 10 min each. Then, the coverslips were mounted in an appropriate anti-fade mounting medium. Fluorescence was observed using an Olympus biological fluorescence microscope (IX2-ILL100, Olympus, Tokyo, Japan).

### 2.3. Cell Proliferation Assay

Cell proliferation was measured by the water-soluble tetrazole-1 (WST-1) assay. The cells, treated with 10 µg/mL recombinant C-reactive protein (CRP) (ab111647, Abcam, Cambridge, MA, USA) and the same volume of Tris-HCl (pH 8.0, containing 20% glycerol, 12.01% urea, 67% Tris-HCl buffer), were plated in 96-well culture plates (1 × 10^3^ per well); WST-1 (Roche, Indianapolis, IN, USA) assay measuring the activity of mitochondrial dehydrogenases was performed following the manufacturer’s instructions at 0-, 1-, 2-, 3-, 4- and 5-day time points.

### 2.4. In Vitro Invasion Assay

For the in vitro invasion assay, similar experiments were performed using inserts coated with a matrigel basement membrane matrix (BD biosciences, Franklin Lakes, NJ, USA). DMEM/F12 containing 15% FBS as a chemoattractant was placed in the lower wells. 200 μL of the synoviocyte suspension containing niclosamide was added to the upper compartments. The chambers were incubated at 37 °C under 5% CO_2_ for 16 h. After incubation, the non-migrating cells were removed from the upper surface of the filter using a cotton swab. The filters were fixed in methanol for 15 min and stained with 0.1% crystal violet for 15 min. Migration was quantitated by counting the stained cells that migrated to the bottom side of the membrane using an optical microscope (100× magnification). All experiments were replicated at least three times with duplicates of each sample.

### 2.5. Flow Cytometry Assay

For the cell-cycle assay, RA–FLS1 and RA-FLS2 cells, treated with or without celastrol, were digested with 2 mM EDTA in PBS and rinsed twice with ice-cold PBS solution, then fixed by adding them dropwise into 75% ice-cold ethanol, followed by incubation in ice for 60 min. The fixed cells were washed with ice-cold PBS and incubated at 37 °C for 30 min in 0.5 mL PBS solution containing 20 mg/mL RNase A, 0.2% Triton X-100, 0.2 mM EDTA and 20 mg/mL of propidium iodide (PI). DNA content was determined by fluorescence-activated cell sorting (FACS) analysis (Becton Dickinson, Franklin Lakes, NJ, USA). The percentage of cells in G0/G1 phases was determined using Flowjo software (Tree Star, Inc., Ashland, OR, USA). For apoptosis assessment by serum withdrawal, cells were washed twice with PBS and then starved by exposure to 1 mL of serum-free culture medium for 48 h. At the end of incubation, the cells were treated as above for detection by flow cytometry.

### 2.6. DNA Microarray

After washing the RA–FLSs with 50 mM potassium phosphate buffer (pH 7.4), the total RNA of each sample was extracted by the RNeasy Mini Kit (Qiagen, Valencia, CA, USA). The procedure for the extraction of the total RNA was according to the manufacturer’s instructions. The quality of the extracted RNA was determined with Bioanalyzer 2100 (Agilent Technologies, Santa Clara, CA, USA). GeneChip^®^ arrays (Affymetrix) were used as the DNA microarrays. DNA microarray analysis was performed with Bio Matrix Research. Statistical analysis after data acquisition and normalization of expression data was performed using GeneSpring (Agilent Technologies, Santa Clara, CA, USA). For the pathway- or function-based category classification, the Munich Information Center for Protein Sequence (MIPS) was used.

### 2.7. RNA Extraction and Real-Time PCR Analysis

Total RNA was isolated from tissues by using AxyPrepTM Blood Total RNA MiniPrep Kit (Axygen) according to the manufacturer’s instructions. First strand cDNA was synthesized with RevertAid First Stand cDNA Synthesis Kit (Fermentas, Waltham, MA, USA) using random hexamer primers. Quantitative PCR was performed through the Bio-Rad (Hercules, CA, USA) Chromo4 real-time PCR system. The primer sets for amplifying targeted genes are listed in Table 1. At the end point of PCR cycles, melt curves were made to check product purity. The levels of targeted genes were expressed as ratios relative to the β-actin mRNA in each sample. Exploratory data analysis using box plots was applied to visually identify the expression level of the target mRNA. Statistical analysis was performed using a chi-square test or Fisher exact test. *p*-Values less than 0.05 were considered statistically significant.

### 2.8. ELISA Assay

Cells were seeded in 6-well plates and cultured in DMEM with 10% FBS for 24 h before treatment. RA–FLSs were treated with celastrol (10 ng/mL) for different times, and the control group cells had no treatment. Cell culture supernatants were then collected. To further study the effect of celastrol on IL-32, MMP-1 and MMP-9 expression in RA–FLS cells treated with IL-1β, the cells were pre-treated with IL-1β for 3 h, and subsequently, different doses (5, 10, 20 and 40 mg/dL) of celastrol (dissolved in anhydrous alcohol) were added to each well. After 24 h, the supernatants were collected for detection of the expression levels of IL-32, MMP-1 and MMP-9 using ELISA. ELISA was performed according to the manufacturer’s instructions. All the experiments were performed in triplicate. Employing commercially available kits, the expression values were calculated on the basis of the standard curve constructed for each assay.

### 2.9. Western Blot Analysis

Protein samples were extracted in RIPA lysis buffer containing protease inhibitors and phosphatase inhibitors (Thermo Scientific, Waltham, MA, USA). 20–40 μg of cell lysate, determined by the BCA protein assay, was separated on 12% SDS–PAGE gels and electrophoretically transferred onto nitrocellulose membranes (Bio-Rad, Hercules, CA, USA). Membranes were incubated with blocking buffer (5% non-fat milk in TBS) for 60 min at room temperature. Then, the membranes were incubated overnight at 4 °C with primary antibodies. The membranes were incubated with secondary antibodies (1:2000, Cell Signaling Technology, Danvers, MA, USA) for 1 h at room temperature. Immuno-reactive bands were visualized by an enhanced chemiluminescence (ECL, Amersham Pharmacia Biotech, Piscataway, NJ, USA) reaction. Each blot is a representative of at least three similar independent experiments.

Subcellular fractionation was performed as previously described [20]. Briefly, cells were lysed in an ice-cold solution containing 0.02% digitonin, 5 mM sodium phosphate (pH 7.4), 50 mM NaCl, 150 mM sucrose, 5 mM KCl, 2 mM dithiothreitol, 1 mM MgCl_2_, 0.5 mM CaCl_2_ and 0.1 mM phenylmethylsulfonyl fluoride. The cytoplasmic fraction was collected after centrifugation of lysates at 1000× *g* for 10 min at 48 °C. The resulting pellet was re-suspended in the lysis solution without digitonin, and loaded onto a cushion of a solution containing 30% w/v sucrose, 2.5 mM Tris-HCl (pH 7.4) and 10 mM NaCl. After centrifugation at 1000× *g* for 10 min at 48 °C, nuclei were collected and extracted for 30 min at 48 °C with an ice-cold solution containing 0.5% *v/v* Triton X-100, 50 mM Tris-HCl (pH 7.5), and 300 mM NaCl. After centrifugation of the extract at 12,000× *g* for 10 min at 48 °C, the supernatant was collected as the nuclear fraction.

### 2.10. Statistical Analysis

All the data were entered into a computer database and analyzed using SPSS 13.0 software (SPSS, Inc., Chicago, IL, USA). The results were expressed as the mean ± standard deviation and statistical comparisons were performed using a completely randomized design analysis of variance and least-significant difference. In all the cases, *p* < 0.05 was considered to indicate a statistically significant difference.

## 3. Results

### 3.1. Isolation and Identification of FLSs from Patients with RA

**Figure 1:**
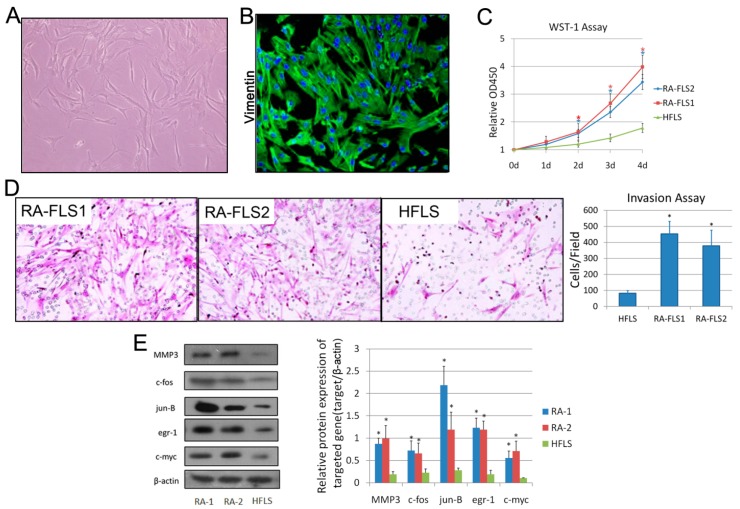
Isolation and identification of fibroblast-like synoviocytes (FLS) from synovial tissues of rheumatoid arthritis (RA) patients. (**A**) Microscope photo of FLSs isolated from human synovial tissue at passage 3 (100×). (**B**) Immunofluorescence analysis of the isolated FLSs using anti-Vimentin antibody and DAPI. (**C**) WST-1 assay, measuring the activity of mitochondrial dehydrogenases, was performed following the manufacturer’s instructions at 0-, 1-, 2-, 3- and 4-day time points. Error bars represent standard deviation (SD) of the mean. * *p* < 0.05 versus the normal human FLS line (HFLS). (**D**) Cell invasion was determined using a Transwell assay as described in the Materials and Methods Section. Microscopic image of migrated cells is shown. Original magnification: 200×. Diagrams of migrating cells from the groups are shown, which were obtained from more than three independent experiments. * *p* < 0.05 versus HFLS. (**E**) Total cell lysates of RA–FLS1 and 2, and HFLS, were examined by Western blotting. Diagrams of the relative gray value are shown, which were obtained from more than three independent experiments. * *p* < 0.05 versus HFLS.

The primary culture of RA synovial cells resulting from trypsin/collagenase digestion contained large, spindle-shaped cells (Figure 1A). Positive expression of vimentin, a marker for FLS [21], was observed in the isolated cells (Figure 1B). The activation levels of two RA–FLS lines (RA–FLS1 and 2) and normal HFLSs were evaluated by various approaches. As shown in Figure 1C,D, the RA–FLSs showed higher proliferation ratios and invasive abilities than the HFLSs. Activation of fibroblasts in vitro is known to generate several functional responses, such as production of matrix components, soluble mediators or enzymes that could contribute significantly to joint pathology in chronic RA in vivo [22,23,24,25]. As shown in Figure 1E, the protein levels of MMP3, c-fos, jun-B, egr-1 and c-myc were abundant in RA–FLSs. These all indicated that RA–FLSs were more activated than normal FLSs.

### 3.2. Treatment of Celastrol Attenuated the Activation Status of RA–FLSs

**Figure 2:**
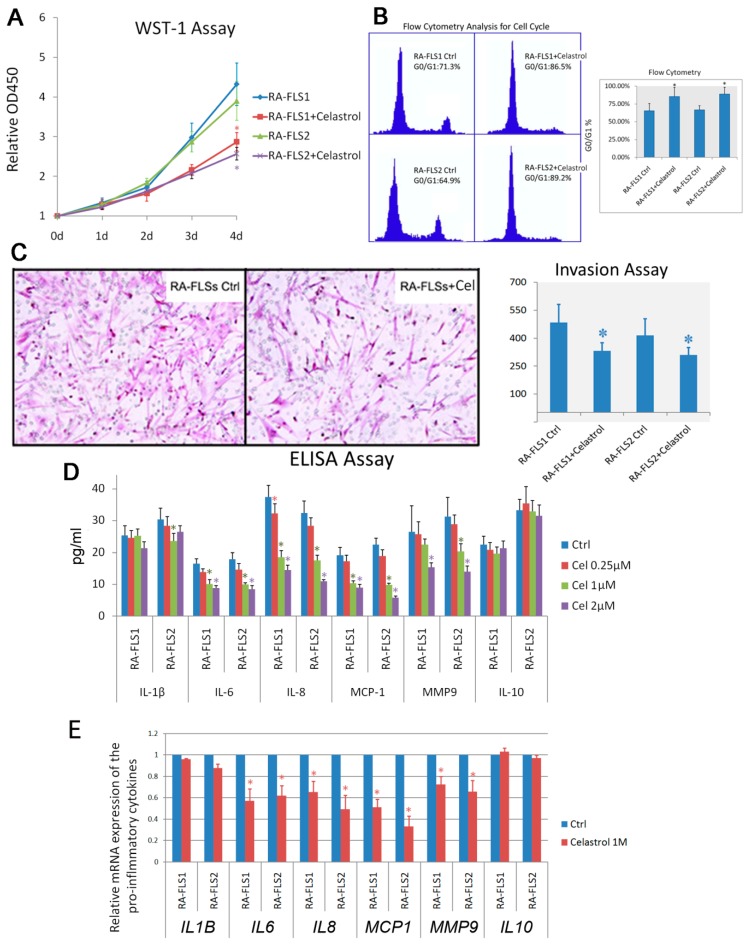
Celastrol-treatment impaired the activation status of RA–FLSs. (**A**) Water-soluble tetrazole-1 (WST-1) assay measuring the activity of mitochondrial dehydrogenases was performed following the manufacturer’s instructions at 0-, 1-, 2-, 3- and 4-day time points. Error bars represent standard deviation of the mean. (**B**) RA–FLS1 and 2 cells were treated with or without celastrol (Cel, 1 μM), and cell-cycle distribution was determined by propidium iodide flow cytometry. Data bars represent the mean absorbance ± SD from triplicate wells from three separate experiments. * *p* < 0.05 versus control. (**C**) Invasion of RA–FLS1 and 2, treated with or without celastrol (1 μM), was determined using a Transwell assay as described in the Materials and Methods Section. Representative figures are shown, and diagrams of migrating cells were obtained from independent approaches. * *p* < 0.05 versus control. (**D**) The release of indicated cytokines was measured using ELISA. Diagrams of the relative cytokine release are shown, which were obtained from more than three independent experiments. * *p* < 0.05 versus control. (**E**) Real-time PCR analysis was carried out to examine the mRNA expression of selected genes. * *p* < 0.05 versus control.

Celastrol is well known for its immuno-suppressive function. Here, we first examined the changes of the biological behaviors of RA–FLSs that were induced by celastrol treatment. After the RA–FLSs were treated with 50 μg/mL celastrol for 24 h, impaired cell proliferation, as well as cell-cycle arrest, were observed (Figure 2A,B). The invasion of the RA–FLSs was also inhibited by celastrol treatment (Figure 2C). To investigate the possible effects of celastrol on pro-inflammatory cytokine secretion, we performed multiplex cytokine assay kits to evaluate the effect of celastrol on the levels of pro-inflammatory mediator productions in culture supernatants of the RA–FLSs. RA–FLS1 and 2 cells were treated with increasing concentrations of celastrol ranging from 0.25 μM to 2 μM for 24 h. The results showed that celastrol significantly reduced the secretion of IL-6, IL-8 and MCP-1 in a dose-dependent manner. We also investigated the effect of celastrol on the production of IL-10, an anti-inflammatory cytokine in RA. It was shown that celastrol did not change the secretion of IL-10 in the RA–FLSs. We also performed real-time PCR analysis to examine the mRNA-level changes of the pro-inflammatory cytokines induced by celastrol treatment. The results were similar to those from ELISA assays.

### 3.3. Expression of Several Chemokine and Chemokine Receptors Was Influenced by Celastrol Treatment in RA–FLSs

**Figure 3:**
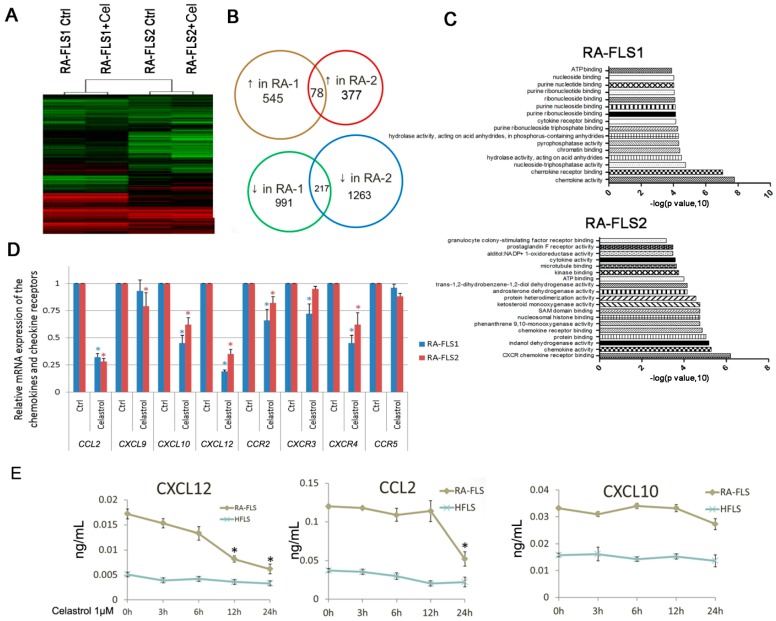
Altered expression of chemokine and chemokine receptors by celastrol treatment in RA–FLSs. (**A**) The heatmap illustrates the genes most significantly influenced by celastrol (1 μM) treatment using microarray analysis. (**B**) The number of the both up- and downregulated genes by celastrol treatment in RA–FLS1 and 2 cells. (**C**) Gene ontology analysis of the most-altered gene groups by celastrol treatment in RA–FLS1 and 2 cells. (**D**) Real-time PCR analysis was carried out to examine the mRNA expression of selected genes screened by microarray analysis. * *p* < 0.05 versus control. (**E**) ELISA analysis was performed to examine the release of the indicated cytokines after celastrol (1 μM) treatment for different times in the RA–FLSs. Data bars represent the mean value ± SD from triplicate wells from three separate experiments using RA–FLS1 and 2 cells. * *p* < 0.05 versus 0 h.

The anti-inflammation effect of celastrol may be mediated by repressing multiple signaling pathways, rather than by targeting one or a few cancer-associated proteins. We used microarray analyses to profile the transcriptional changes by celastrol treatment in the RA–FLSs (Figure 3A). It was found that celastrol treatment resulted in 545 upregulated and 991 downregulated genes in RA–FLS1, and 377 up- and 1263 downregulated genes in RA–FLS2. Among these genes, 78 up- and 217 downregulated genes were shared in both FLS strains (Figure 3B, Supplementary Table S2). The gene ontology analysis showed that the expression of some chemokines and chemokine receptors was altered significantly after celastrol treatment (Figure 3C).

The qRT–PCR analysis was performed to confirm the expression levels of differentially expressed chemokines, chemokine receptors as well as other related inflammatory cytokines. As shown in Figure 3D, mRNA levels of *CCL2*, *CXCL10*, *CXCL12*, *CCR2*, *CXCR3* and *CXCR4* were significantly downregulated in celastrol-treated cells. On the other hand, the altered expression of *IL-6*, *IL-16* and *MMP9* was also confirmed by qRT–PCR (Supplementary Figure S1), which was in accordance with previous findings.

Examination of the release of CCL2, CXCL10 and CXCL12, using the ELISA essay, showed that celastrol suppressed the release of CXCL12 in a time-dependent way, while had a late effect on CCL2, and little effect on CXCL10 (Figure 3E).

### 3.4. NF-κB Signaling Pathway Was Partially Involved in the Celastrol-Mediated Regulation of CCL2 and CXCL12

**Figure 4:**
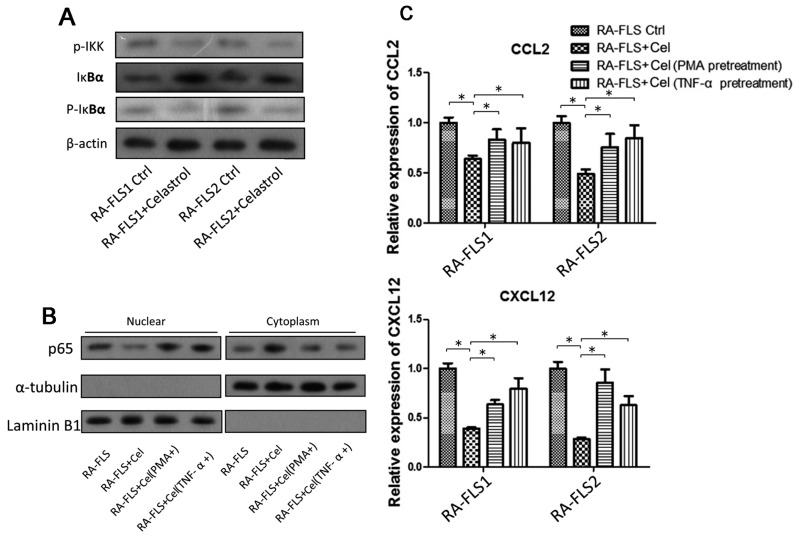
NF-κB signaling pathway might be involved in celastrol-suppressed chemokine production. (**A**) Total cell lysates of RA–FLS1 and 2 cells treated with or without celastrol (1 μM) were examined by Western blotting. (**B**) Subcellular fractionation was performed as described in the Materials and Methods Section; nuclear and cytoplasmic lysates of RA–FLSs with or without celastrol treatment were examined by Western blotting. (**C**) After RA–FLS1 and 2 cells were treated with celastrol (1 μM) or celastrol (1 μM) together with pretreatment of two NF-κB activators (PMA and TNF-α), real-time PCR analysis was carried out to examine the mRNA expression of CCL2 and CXCL12. Data bars represent the mean value ± SD from triplicate wells from three separate experiments using RA–FLS1 and 2 cells. * *p* < 0.05 versus RA-FLS1/2 treated with celastrol.

Previous findings have shown that celastrol could significantly suppress the activation of the nuclear factor kappa B (NF-κΒ) signaling pathway in RA–FLS. The NF-κΒ signaling pathway is associated with the regulation of IL6, IL-8 and MMP9. In the present study, we also showed that treatment of celastrol on RA–FLSs could reduce the phosphorylation of IKK and IKBα (Figure 4A), and inhibit the translocation of NF-κΒ p65 from the cytoplasm to nucleus (Figure 4B).

We wondered whether the expression of chemokines was regulated by the same NF-κΒ signaling pathway that was influenced by celastrol treatment. Two activators of NF-κΒ signaling, PMA and TNFα, were used here. As shown in Figure 4C, activation of NF-κΒ could partially reverse the celastrol-mediated downregulation of CCL2 and CXCL12 (Figure 4C). These indicated that the NF-κΒ signaling pathway was at least partially involved in the celastrol-mediated inhibition of chemokine production, which was a novel finding. On the other hand, it was found that the p38/MAPK pathway also played a minor role in the celastrol-regulated chemokine expression (data not shown here).

## 4. Discussion

Activated FLSs in the synovium of RA patients have been reported in various studies. Celastrol showed a convincing therapeutic effect in a clinical trial of RA and an animal model of arthritis. However, there is very limited data about the influence of celastrol treatment on activated RA–FLSs in vitro. In the present study, we investigated the effect of celastrol on RA–FLSs, and performed a high-throughput approach for the screening of the genes regulated by celastrol in RA–FLSs.

The activation status of RA–FLSs was high in RA patients but lower in RA cell lines. Here, we isolated and purified FLSs from two synovial tissues from RA patients for in vitro studies. We showed that celastrol treatment could markedly modulate the activation status of RA–FLSs, such as via attenuation of both the proliferation and invasion of the activated RA–FLSs, which provided more evidence of the potential role of celastrol treatment on RA. This is the first report on the gene expression profiles regulated by celastrol in RA–FLSs. The results from the gene ontology analysis showed that gene expression of specific chemokines, chemokine receptors and those involved in chemokine activity, including CCL2, CXCL10, CXCL12, CCR2 and CXCR4, was significantly reduced by celastrol treatment in RA–FLSs, which is a novel finding.

Chemokines promote leukocyte infiltration and activation, angiogenesis, osteoclast differentiation, and synoviocyte proliferation and activation, and participate in the generation of pain by regulating the release of neurotransmitters. A number of chemokines are expressed in a timely controlled fashion in the joint during arthropathies, regulating all the aspects of inflammation as well as the equilibrium between damage and repair and between relief and pain. Here, we showed the possible involvement of CCL2, CXCL10, CXCL12, CCR2 and CXCR4 in the progression of RA mediated by activated FLSs. Targeting of these specific chemokine/chemokine receptor interactions might be a useful tool for therapeutic intervention.

In addition, we showed that release of CCL2 and CXCL12 proteins from the RA–FLSs was downregulated significantly by celastrol treatment. To further confirm this, we also applied other two RA–FLS lines (RA–FLS3/4) derived from RA patients, which were not so highly activated as RA–FLS1/2 (Supplementary Figure S2). It was found that the treatment of celastrol could also attenuate the release of CCL2 and CXCL12 (Supplementary Figure S3). This provides new evidence of the molecular mechanism of celastrol’s anti-inflammatory effect.

Activation of the NF-κB signaling pathway could mitigate the effects of downregulation of CCL2 and CXCL12 in RA–FLSs by celastrol treatment, indicating that NF-κΒ is upstream of these two genes. The NF-κB signaling pathway was strongly modulated by celastrol treatment in RA–FLS. In particular, celastrol treatment attenuated the activation and translocation of NF-κB p65. NF-κB p65 signaling is known to be involved in the regulation of many cytokines, adhesion molecules, chemokines, receptors and adaptive enzymes [26,27,28,29]. Thus, celastrol mediated its immune-suppressive activity, at least partially, through the NF-κB signaling.

Moreover, we found that the NF-κB p65 pathway was also involved in the celastrol-suppressed IL6, IL8 and MMP9 production, but had only a minor influence on the production of MMP3 (Supplementary Figure S4). Thus, celastrol might also play anti-inflammatory roles in RA through other signaling pathways, such as the JNK and ERK pathways. This also led to the downregulation of CCL2 and CXCL12.

In summary, our results demonstrate that celastrol treatment significantly influences the expression of chemokine and chemokine receptors in RA–FLSs, which might be a partial molecular mechanism of its therapeutic effect. However, further underlying mechanisms and side effects of this herbal extract need to be investigated in the future.

## Figures and Tables

**Table 1:** Primer sets used for quantitative real time PCR (qRT–PCR).

Gene	Forward	Reverse
*IL-1β*	GCTTATTACAGTGGCAATGAGGAT	TAGTGGTGGTCGGAGATTCG
*IL-6*	GCCACTCACCTCTTCAGAAC	GCAAGTCTCCTCATTGAATCCA
*IL-8*	AGGACAAGAGCCAGGAAGAA	GGGTGGAAAGGTTTGGAGTATG
*CCL2*	CTGTGCCTGCTGCTCATAG	CTTGCTGCTGGTGATTCTTCT
*MMP-3*	CAGCAAGGCATAGAGACAACAT	CGCACAGCAACAGTAGGATT
*IL-10*	GCCAAGCCTTGTCTGAGATG	GCATTCTTCACCTGCTCCAC
*CXCL9*	CCCTGTTTCTTCCACAGTGC	GCACCTGCTCTGAGACAATG
*CXCL10*	AAGGATGGACCACACAGAGG	AGTAGCAGCTGATTTGGTGAC
*CXCL12*	TGGGCACATTGATCTGGGAT	CAGGTACAGGGCATGGATGA
*CCR2*	CAGTTGCTGAGAAGCCTGAC	AGAACGAGATGTGGACAGCA
*CXCR3*	AGGTGCCCTCTTCAACATCA	GCTGGGTGGCATGAACTATG
*CXCR4*	GAGGCCCTAGCTTTCTTCCA	GAATGTCCACCTCGCTTTCC
*CCR5*	CTTCTGGGCTCCCTACAACA	GTCACCTGCATAGCTTGGTC
*ACTB*	GACCTGACTGACTACCTCATGAAGAT	GTCACACTTCATGATGGAGTTGAAGG

## Supplementary Materials

The following are available online at www.mdpi.com/2073-4425/8/9/221/s1. Figure S1 Real-time PCR analysis was carried out to examine the mRNA expression of selected genes after RA–FLS1 and RA–FLS2 were treated with or without celastrol (1 μM). Figure S2 Left, WST-1 assay measuring the activity of mitochondrial dehydrogenases was performed following the manufacturer’s instructions at 0-, 1-, 2-, 3- and 4-day time points. Error bars represent standard deviation of the mean. * *p* < 0.05 versus HFLS. Right, total cell lysates of RA–FLS1, 2 and HFLS were examined by Western blotting. Diagrams of the relative gray value are shown, which were obtained from more than three independent experiments. * *p* < 0.05 versus HFLS. Figure S3 Real-time PCR analysis was carried out to examine the mRNA expression of selected genes. * *p* < 0.05 versus control. Figure S4. After RA–FLS1 and 2 cells were treated with WA or WA together with pretreatment of two NF-κB activators (PMA and TNF-α), real-time PCR analysis was carried out to examine the mRNA expression of IL6, IL8, MMP3 and MMP9. Table S1. Demographic and disease characteristics of the RA patients included in the present study. Table S2. The list of genes whose expressions were altered after treatment of celastrol.
