# Supplementary material for: High-Throughput Study of the Effects of Celastrol on Activated Fibroblast-Like Synoviocytes from Patients with Rheumatoid Arthritis
Source: Genes (Basel). 2017 Sep 6;8(9):221. doi: 10.3390/genes8090221 (PMC5615354; doi:10.3390/genes8090221)
Supplement: Supplementary file 1 [file genes-08-00221-s001.zip › Supplementary Table S2.docx]

| **Supplementary Table S1: Demographic and desease characteristics of the RA patients included in the present study** | | |
| --- | --- | --- |
|  | RA1 | RA2 |
| Age of onset | 29 | 50 |
| Gender | Female | Female |
| Disease duration | 30years | 16 |
| Tender joint count | 26 | 18 |
| Swollen joint count | 20 | 18 |
| MHAQ | 3 | 2.25 |
| CRP (mg/dl) | 36 | 113 |
| ESR (mm/h) | 80 | 70 |
| DAS28-CRP | 8.32 | 7.15 |
| PtPainVAS | 8 | 7 |
| PtGVAS | 8 | 7 |
| DrGVAS | 8 | 7 |
| Use of MTX | Yes | Yes |
| Use of corticosteroid | Yes | Yes |
| Use of biologics | No | No |
