# Supplementary figures and images for: High-Throughput Study of the Effects of Celastrol on Activated Fibroblast-Like Synoviocytes from Patients with Rheumatoid Arthritis
Source: Genes (Basel). 2017 Sep 6;8(9):221. doi: 10.3390/genes8090221 (PMC5615354; doi:10.3390/genes8090221)

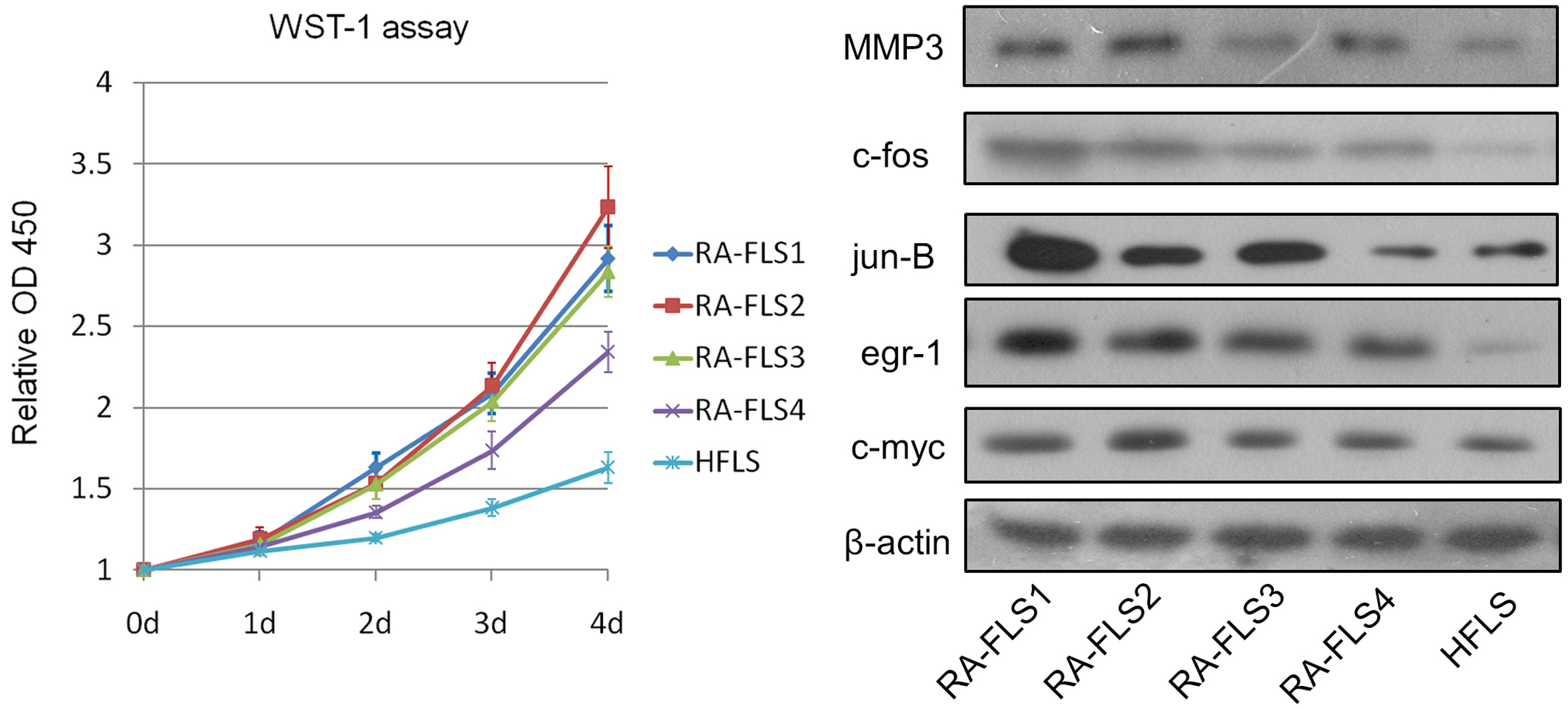

Supplement: Supplementary file 1 [file genes-08-00221-s001.zip › Figure S2.tif]

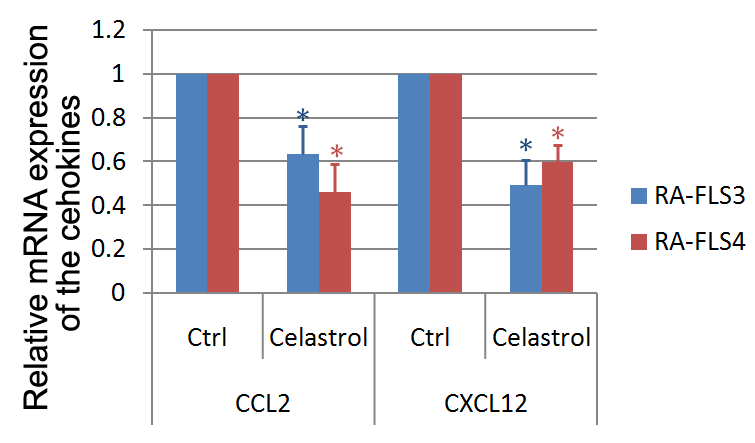

Supplement: Supplementary file 1 [file genes-08-00221-s001.zip › Figure S3.tif]

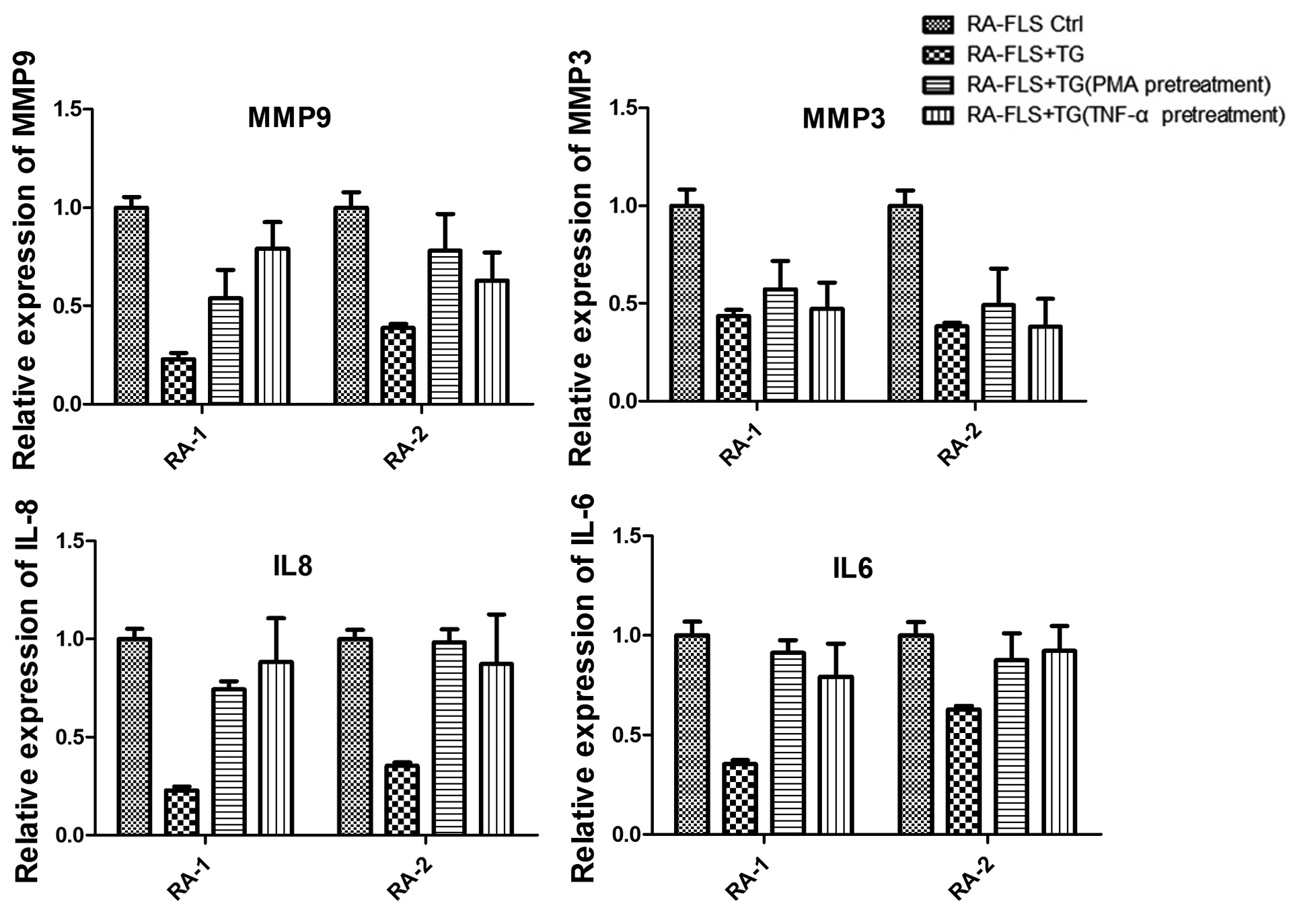

Supplement: Supplementary file 1 [file genes-08-00221-s001.zip › Figure S4.tif]

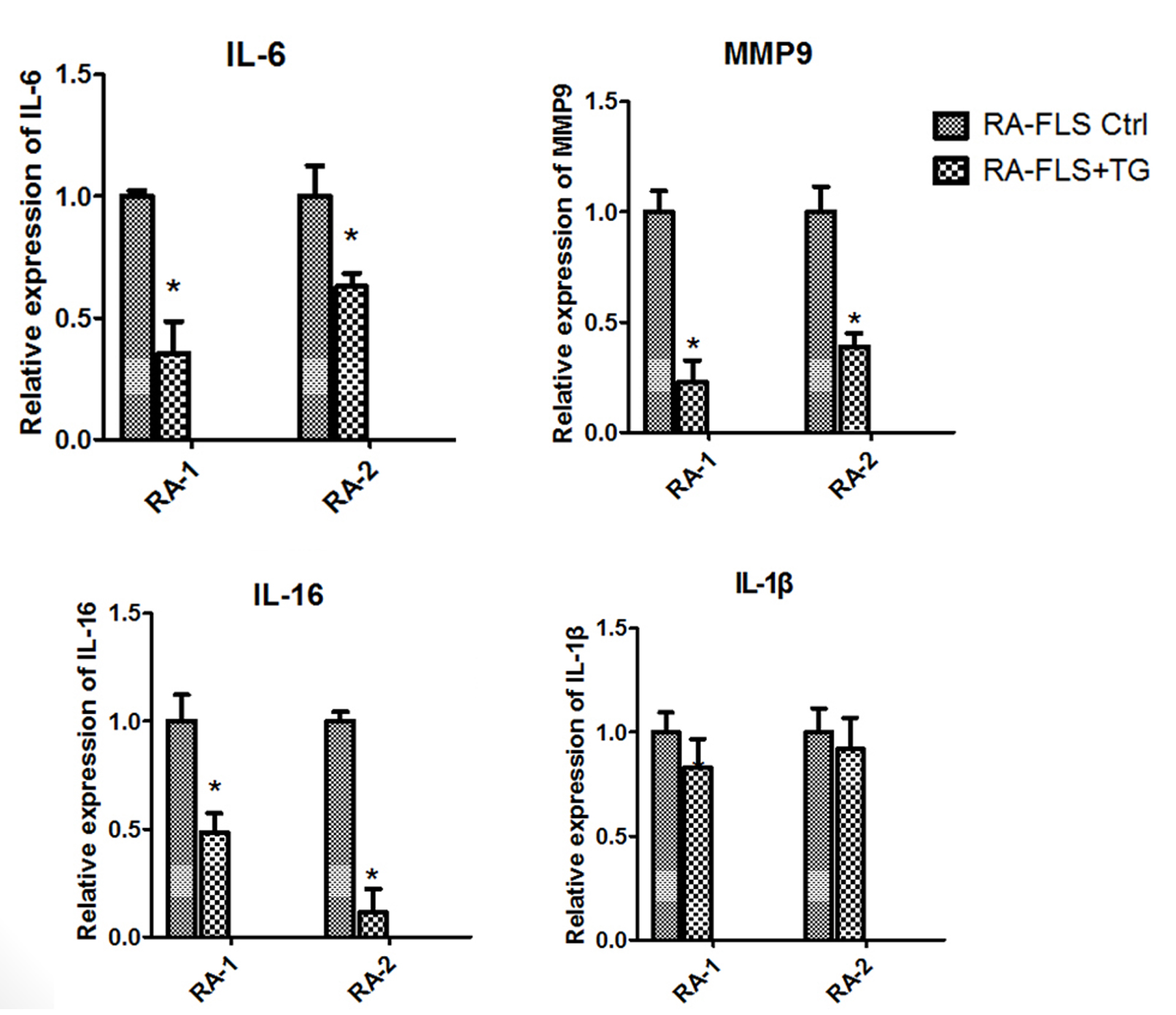

Supplement: Supplementary file 1 [file genes-08-00221-s001.zip › Figure S1.tif]
